# Cancer treatment induces neuroinflammation and behavioral deficits in mice

**DOI:** 10.3389/fnbeh.2022.1067298

**Published:** 2023-01-09

**Authors:** Kimberly Demos-Davies, Jessica Lawrence, Allison Rogich, Erin Lind, Davis Seelig

**Affiliations:** ^1^Department of Veterinary Clinical Sciences, University of Minnesota College of Veterinary Medicine, Saint Paul, MN, United States; ^2^Masonic Cancer Center, University of Minnesota, Minneapolis, MN, United States; ^3^Department of Neuroscience, University of Minnesota, Minneapolis, MN, United States

**Keywords:** cancer treatment, neuroinflammation, neurobehavior, cancer-related cognitive impairment, SKH1 mice

## Abstract

**Introduction:** Cancer survivors are increasingly diagnosed with a syndrome of neurocognitive dysfunction termed cancer-related cognitive impairment (CRCI). Chemotherapy and radiation therapy have been implicated in CRCI; however, its underlying pathogenesis remains unclear, hindering effective prevention or treatment.

**Methods:** We used the hairless strain SKH1 (11–12-week-old) and treated the mice with radiation to the right hindlimb, doxorubicin (a chemotherapy agent), concurrent radiation, and doxorubicin, or no treatment (control). Neurocognition was evaluated *via* standardized behavioral testing following treatment. Mice were subsequently humanely euthanized, and plasma and brains were collected to identify inflammatory changes.

**Results:** Mice treated with radiation, doxorubicin, or both radiation and doxorubicin demonstrated equivalent hippocampal dependent memory deficits and significant increases in activated microglia and astrocytes compared to control mice. Doxorubicin-treated mice had significantly increased plasma IL-6 and failed to gain weight compared to control mice over the study period.

**Discussion:** This study demonstrates that non-brain directed radiation induces both gliosis and neurocognitive deficits. Moreover, this work presents the first characterization of SKH1 mice as a relevant and facile animal model of CRCI. This study provides a platform from which to build further studies to identify potential key targets that contribute to CRCI such that strategies can be developed to mitigate unintended neuropathologic consequences associated with anticancer treatment.

## Introduction

Advancements in cancer therapy have improved cure rates and increased the life span of cancer patients (Feiock et al., 2016). This increased longevity has resulted in the recognition of treatment-related, long-term sequelae, including cancer-related cognitive impairment (CRCI; Feiock et al., 2016; Santos and Pyter, 2018). CRCI is a syndrome of neurocognitive dysfunction characterized by learning and memory deficits, alterations of attention, concentration, processing speed, and executive function (Feiock et al., 2016). It is estimated that over 75% of cancer survivors, or over 3.9 million people, will develop CRCI with symptoms that disrupt quality of life for up to 10 years following treatment (Janelsins et al., 2011; Seigers et al., 2015; Feiock et al., 2016). CRCI decreases quality-of-life measures through restricted functional independence, thus negatively impacting the survivor’s social, family, and professional life (Janelsins et al., 2011; Seigers and Fardell, 2011; Feiock et al., 2016). A potential life-threatening consequence of CRCI ha decreased compliance to monitoring schedules that are intended to diagnose early recurrence or metastasis, which may decrease long-term outcome (Janelsins et al., 2011; Feiock et al., 2016). Chemotherapy and radiation therapy have both been implicated in inducing CRCI following treatment for many solid tumors, including breast, lung, intestinal, ovarian, prostatic, and testicular tumors (Argyriou et al., 2011; Hutchinson et al., 2012; Feiock et al., 2016; Santos and Pyter, 2018).

CRCI is well documented in breast cancer survivors, who are commonly prescribed adjuvant chemotherapy and radiotherapy (McDonald et al., 2010; Feiock et al., 2016; Lange et al., 2019). Over 50% of breast cancer survivors have self-reported impairment in working memory, executive function, and processing speed (McDonald et al., 2010; Seigers et al., 2015; Feiock et al., 2016). In female breast cancer patients treated with surgery and chemotherapy, MRI brain studies have revealed changes in gray matter, reductions in white matter microstructure, and alterations in brain activation and connectivity 1 year following the completion of chemotherapy (McDonald et al., 2010; Wefel et al., 2015; Lange et al., 2019).

Research on CRCI suggests that some chemotherapeutic drugs produce reactive oxygen species and inflammatory cytokines that disrupt the blood-brain barrier (BBB) and activate microglia which may lead to neurocognitive changes (Santos and Pyter, 2018; Ren et al., 2019). Chemotherapy agents can also cause neuronal damage through BBB disruption, neuroinflammation, oxidative stress, myelin degradation, impaired neurogenesis, production of damage associated molecular patterns, and changes in brain blood flow (Argyriou et al., 2011; Feiock et al., 2016; Santos and Pyter, 2018; Ren et al., 2019).

The effects of direct radiation therapy on the brain include behavioral abnormalities and neuroinflammation (Feiock et al., 2016; Santos and Pyter, 2018). However, radiation therapy also causes local and distant bystander effects on unirradiated tissue (Wang et al., 2018; Siva et al., 2019). Detrimental bystander effects of radiation treatment include DNA damage in unirradiated cells, reduced unirradiated cell survival, increased circulating cytokine levels, systemic toxicities (nausea, malaise, anorexia), and neurological clinical signs (memory loss, fatigue, impaired concentration; Back et al., 2011; Cho and Kim, 2012; Feiock et al., 2016; Keeney et al., 2018; Santos and Pyter, 2018; Wang et al., 2018; Ren et al., 2019; Siva et al., 2019). To date, only one *in vivo* study has demonstrated the induction of distant and widespread neuroinflammation following hindlimb irradiation that was comparable to changes in mice treated with single agent chemotherapy (Feiock et al., 2016). Despite these observations, little is known about how the indirect effects of radiation might contribute to the cognitive impairment of CRCI.

A solid understanding of the underlying mechanisms of anticancer treatment-related CRCI warrants investigation to increase the likelihood of effective prevention or mitigation. The objective of this study was to investigate the neuroinflammatory and cognitive impact of cancer therapies in non-tumor bearing mice. Our primary hypothesis was that mice treated with chemotherapy or radiation alone would have increased activation of astrocytes and microglia (components of neuroinflammation) with subsequent cognitive impairment compared to control mice (Streit et al., 2004). The second hypothesis was that chemo-radiation would induce greater neuroinflammation and cognitive dysfunction compared to chemotherapy or radiation alone.

## Materials and Methods

### Experimental animals

Experiments were approved by and performed in accordance with the University of Minnesota Institutional Animal Care and Use Committee (IACUC). Female SKH1 mice were purchased from Charles River Laboratories (Wilmington, MA). The SKH1 mouse was selected because this strain has been previously used, by us and others, to evaluate the detrimental effects of extracranial radiation (Benavides et al., 2009; Cho and Kim, 2012). Female SKH1 mice were evaluated in this study as CRCI is overrepresented in female breast cancer patients, and because women have been shown to have greater cognitive impairment than men following surgery for early-stage colorectal carcinoma (Vardy et al., 2014; Santos and Pyter, 2018). Moreover, female mice have been shown to have less unstructured variance in temperature and activity compared to males, reducing potential uncertainty in behavior during testing within the study groups (Smarr and Kriegsfeld, 2022). Non-tumor bearing mice were selected to isolate treatment-related changes from tumor changes as cancer is known to cause cognitive changes (Janelsins et al., 2011; Santos and Pyter, 2018; Lange et al., 2019).

The mice were divided into four groups based on body weight. Groups were defined as mice treated with doxorubicin (DOX), extracranial hindlimb radiation treatment (RT), both doxorubicin, and radiation (DOX-RT) or anesthesia only (Control) groups. The time period for the study was chosen based on consultation with the University of Minnesota Mouse Behavior Core and literature review of chemotherapy only treated mice studies. Mice were 11–12-week old at the start of the study. Mice were weighed on the day of treatment and every other day for the duration of the 16-day study period. Mice were euthanized by carbon dioxide proceeded by exsanguination following the University of Minnesota IACUC Criteria for Carbon Dioxide Euthanasia Guidelines.

### Doxorubicin (DOX)

To evaluate the cognitive impact of systemic chemotherapy, doxorubicin was selected as it is the base drug for standard of care for breast carcinoma treatment and has been commonly utilized in previous studies examining the cognitive impact of chemotherapy (McDonald et al., 2010). Doxorubicin HCL (Hikma Pharmaceuticals USA Inc., Berkeley Heights, NJ, USA) was purchased from the University of Minnesota Boynton Health Pharmacy. Doxorubicin was administered by single 5 mg/kg intraperitoneal (IP) injection either alone or concurrently with radiation treatment. The route of administration and dose of doxorubicin was previously reported to be safe in immunocompetent mice (Keeney et al., 2018). The IP route was elected because of ease of administration, and because plasma concentrations in nude mice are similar following IP or intravenous administration (Johansen, 1981). Because doxorubicin is a radiosensitizer and concurrent doxorubicin at the standard dose (60 mg/m^2^) and irradiation in humans may lead to unacceptable toxicity, we elected to use a single dose of 5 mg/kg (equivalent to 18 mg/m^2^), just below the postulated acute lethal dose in mice of 7–10 mg/kg IP (Johansen, 1981; Ismaili et al., 2010). This is similar to studies in human cancer patients when concomitant doxorubicin is administered (10–20 mg/m^2^ weekly) during radiation therapy (Pisters et al., 2004; Romesser et al., 2021). Control mice were treated with saline IP with the volume matched to that of doxorubicin in the chemotherapy-treated mice.

### Radiation treatment (RT)

Mice were prescribed a dose of 20 Gy radiation to the skin surface of the right hindlimb using 6 MeV electrons (Varian 2100 iX; Varian Medical Systems, Inc., Palo Alto, CA) and a custom 2 × 2 cm cutout. Tissue equivalent bolus (1 cm) was placed on the surface of the skin to provide sufficient dose build-up to the level of the skin. The proximal edge of the field was palpated to ensure the caudal spine was not within the treatment field. Mice were anesthetized with ketamine (90 mg/kg) and xylazine (4 mg/kg) administered IP to ensure immobilization for treatment. Live video monitoring of each mouse was used to monitor positioning and anesthesia during treatment. Radiation dose at the level of the irradiated skin on the right hindlimb, unirradiated skin on the left hindlimb, and skin over the calvarium (indirect measure of brain exposure) was quantified *via* radiochromic film dosimetry (GAFchromic^TM^ EBT2, Ashland Advanced Materials, Bridgewater, NJ) to verify the prescribed dose was as expected and that dose was undetectable in non-target sites (skin over the skull; Baghani et al., 2015). Control mice were anesthetized with xylazine and ketamine as described for irradiated mice.

### Open field test

**Figure 1:**
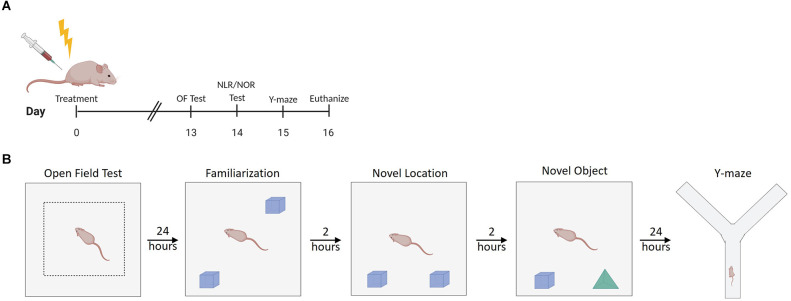
Experimental design summary. **(A)** Experimental timeline. **(B)** Illustration of setup characteristics for each behavior test, including familiarization with objects prior to NLR/NOR testing. OF Test, Open Field Test; NLR, Novel Location Recognition Test; NOR, Novel Object Recognition Test; Y-maze, Spontaneous Alternation y-maze. Created with BioRender.com.

Mice were assimilated to the testing room for 30 min before testing. The open field test evaluates the general motor function and anxiety-like behavior (Seigers et al., 2015). General motor function was evaluated by the distance traveled during the open field test. Anxiety-like behavior was measured by the time spent in the middle of the maze (Seigers et al., 2015). The open field test (Figure 1A) was performed 13 days after treatment in a 40 cm × 40 cm gray arena with light intensity in the middle of the arena at 100 lux. Mouse movement was video recorded for 10 min once the mouse was placed in the arena. ANY-maze 6.3 (1999–2020 Stoelting Co., Wood Dale, IL) tracking software was used to record mouse movement and distance traveled during the 10-min period. Mice were subsequently placed back into their home cage.

### Novel location recognition test and novel object recognition test

Mice were assimilated to the testing room for 30 min before familiarization, novel location recognition (NLR), and novel object recognition (NOR) testing. The NLR test evaluates spatial learning and memory (Seigers et al., 2015). The NOR test evaluates recognition memory (Seigers et al., 2015). The familiarization test (Figure 1B) was performed 24 h after the open field test in the same 40 cm × 40 cm gray square arena with light intensity in the middle of the arena at 60 lux. Two of the same objects (Legos, pyramid shaped, dimensions ~3.2 cm × 3.2 cm × 2.9 cm, The Lego Group, Billund, Denmark) were placed in the arena with removable mounting tabs (Scotch, 3M, St Paul, MN). Mouse movement was recorded for 5 min once each mouse was placed in the arena. Mice were subsequently placed back into their home cage. For the NLR test, one of the objects was moved to a new location within the arena, and mice were placed into the arena 2 h after the familiarization test (Figure 1B). Mouse movement was recorded for 5 min once the mouse was placed in the arena. Mice were then placed back into their home cage. For the NOR test, one of the objects was removed and replaced with a novel object (different colored Legos in a tower shape, dimensions ~3.2 cm × 3.2 cm × 3.8 cm) that the mice had never explored before. The mice were placed into the arena 2 h after the NLR test (Figure 1B). Mouse movement was recorded for 5 min once the mouse was placed in the arena. Mice were placed back into their home cage. ANY-maze 6.3 (1999–2020 Stoelting Co., Wood Dale, IL) tracking software was used to record mouse movement for all 5-min test periods. The discrimination ratio was calculated for the NLR and NOR tests using the following formula: discrimination ratio = (time investigating changed object − time spent investigating unchanged object)/(time spent investigating changed object + time spent investigating unchanged object; Antunes and Biala, 2012). This ratio ranges from 1 to −1. A positive ratio indicates the mouse spent more time investigating the changed object than the unchanged object. A negative ratio indicates the mouse spent more time investigating the unchanged object compared to the changed object. A score of 0 indicates chance or null preference (Antunes and Biala, 2012).

### Spontaneous alternation y-maze

Mice were assimilated to the testing room for 30 min before testing. The spontaneous alternation y-maze evaluates hippocampal and prefrontal cortex-dependent working memory (Kraeuter et al., 2019). The spontaneous alternation y-maze was performed in a maze made of an extruded PVC board with visible spatial cues at the end of each of the arms of the maze with a light intensity of 60 lux in the center of the maze. The spontaneous alternation y-maze was performed 24 h after the novel location recognition test, or 15 days post-treatment (Figure 1B). Mouse movement was video recorded for 5 min once the mouse was placed into one arm of the maze. ANY-maze 6.3 (1999–2020 Stoelting Co., Wood Dale, IL) tracking software was used to record mouse movement and the number of arm entries during the 5-min session. The number of alternations was determined by counting the number of times the mice went down all three arms consecutively. The spontaneous alternation percentage was calculated using the following formula: spontaneous alternation percentage = number of alternations/(total number arm entries − 2) × 100 (Kraeuter et al., 2019).

### Immunohistochemistry (IHC)

Collected brains were fixed in 10% neutral-buffered formalin and paraffin-embedded. Five-micron tissue sections were deparaffinized in xylene and then rehydrated in graded alcohol. Antigen retrieval took place in a Biocare Decloaking Chamber (Biocare Medical, Concord, CA) at 80°C for 1 h with sodium citrate buffer pH 6. Tissue sections were blocked with Biocare Background Punisher (Biocare Medical, Concord, CA, 50-823-79) for 30 min in a humidity chamber at room temperature. Sections were immunostained for activated microglia (rabbit polyclonal anti-Iba1; Abcam, Cambridge, MA; 1:100 in TBS) overnight, and activated astrocytes (rabbit polyclonal anti-GFAP; Abcam, Cambridge, MA; 1:2,500 in PBS) for 2 h. Tissue sections were subsequently stained with a secondary antibody, goat anti-rabbit Alexa-Fluor 488 (Invitrogen, Molecular Probes, Inc., Eugene, OR; 1:250 in TBS). Slides were stained, omitting the primary antibody for negative controls. Slides were counterstained with 4’,6-Diamidino-2-Phenylindole, Dihydrochloride (DAPI, Invitrogen, Molecular Probes, Inc., Eugene, OR; 1:500 in PBS) for 20 min in the dark.

### Histological analysis

Seven regions of the brain were analyzed: the caudal cortex, cerebellum, hippocampus, medulla, midbrain, rostral cortex, and striatum. These regions of the brain have been shown to be affected by cancer treatment (McDonald et al., 2010; De Ruiter et al., 2012; Pomykala et al., 2013; Feiock et al., 2016). These regions of the brain were identified using the Allen Reference Atlas (Dong, 2008). The caudal cortex was identified through coronal sections near −3.38 to −4.08 Bregma (Dong, 2008). The cerebellum was identified through coronal sections near −6.355 to −7.255 Bregma (Dong, 2008). The hippocampus was identified through coronal sections near −2.78 to −3.455 Bregma including the dentate gyrus, CA1, CA3 (Dong, 2008). The medulla was identified through coronal sections near −6.355 to −7.255 Bregma (Dong, 2008). The midbrain was identified through coronal sections near −3.38 to −4.08 Bregma (Dong, 2008). The rostral cortex was identified through coronal sections near −0.08 to −1.455 Bregma (Dong, 2008). The striatum was identified through coronal sections near −0.08 to −1.455 Bregma (Dong, 2008). Using an upright microscope (Olympus BX53 microscope with Olympus DP73 camera, Olympus America Inc., Center Valley, PA), 4–5 consecutive, non-overlapping, adjacent images were taken at 200× magnification on DAPI filter and fluorescein isothiocyanate (FITC) filter. The corresponding DAPI and FITC images were overlaid using Photoshop 2021 version 22.5.1 (Adobe, San Jose, CA) and then analyzed using ImageJ (ImageJ 1.53e, Wayne Rashband, and contributors, National Institutes of Health, USA; Schneider et al., 2012). Iba1+ cells and GFAP+ cells were manually counted using the multi-point tool to evaluate microgliosis and astrocytosis, respectively, as components of neuroinflammation (Streit et al., 2004; Feiock et al., 2016; Costa et al., 2018).

### Cytokine analysis

Blood was collected *via* intracardiac puncture and placed into K3 EDTA blood tubes (Strategic Applications Inc., Lake Villa, IL). Following the manufacture protocol, the BD Cytometric Bead Array (CBA) Mouse Inflammation Kit (BD Bioscience, San Jose, CA) and BD FACSCelesta flow cytometer were used to quantify IL-6, IL-10, IL12p70, IFN-γ, MCP-1, and TNF in each mouse. The data was analyzed using the Flowjo CBA plugin (BD Bioscience, San Jose, CA).

### Statistical analysis

Data were presented and analyzed using Prism 8.0 (GraphPad Software, San Diego, CA). One-way analysis of variance (ANOVA) with the Tukey *post-hoc* test was used to evaluate differences between groups for behavioral tests, IHC, and cytokine analysis. Student *t*-tests were used to determine differences in discrimination ratio between groups and chance for NLR and NOR tests. For the discrimination ratio, the chance was defined as a discrimination ratio of 0. Two-way ANOVA with the Tukey *post-hoc* test was used to determine differences in body weight change over time between treatment groups. Significance was set at a *p*-value < 0.05 with one asterisk (*) representing *p* value < 0.05 and two asterisks (**) representing *p*-value < 0.01.

## Results

### Doxorubicin treatment causes significant weight loss over time compared to control groups

Doxorubicin, whether it was given as a single agent or in conjunction with irradiation, induced a significant decrease in body weight change over time compared to control mice (Supplementary Figure 1). DOX-treated mice failed to gain weight over the 16 days of the study. There was no difference in body weight change over time between the mice that underwent radiation alone and the control mice.

### Doxorubicin, hindlimb irradiation, or concurrent doxorubicin and hindlimb irradiation causes hippocampal dependent memory deficits in SKH1 mice

**Figure 2:**
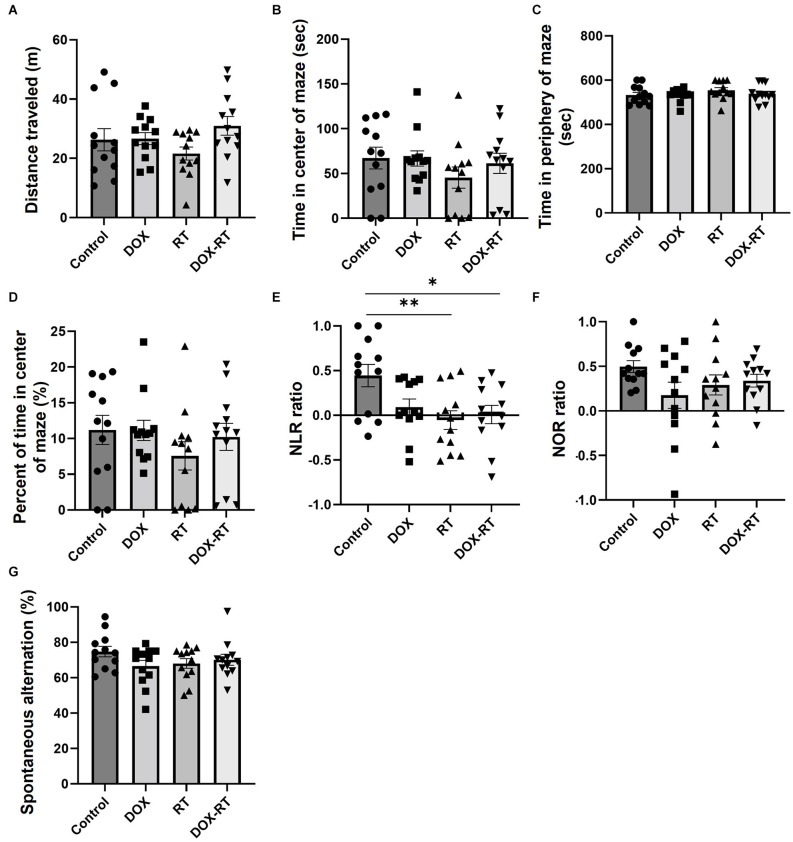
Cancer treatment causes hippocampal dependent memory deficit in mice. **(A)** Open field test did not detect a difference between groups in the total distance traveled. **(B)** Open field test did not detect a difference between groups in time spent in the center of the maze. **(C)** Open field test did not detect a difference between groups in time spent in the periphery of the maze. **(D)** Open field test did not detect a difference between groups in percentage of time spent in the center of the maze. **(E)** Novel location testing showed that RT and DOX-RT mice had a significantly lower discrimination ratio than control mice. **(F)** Novel object recognition test did not detect a difference in discrimination ratio between groups. **(G)** Spontaneous alternation y-maze testing showed a similar percentage of alternations between groups. Data represent the mean and SEM (*n* = 12 per group). DOX, doxorubicin group, RT, hindlimb radiation group, DOX-RT, doxorubicin and hindlimb radiation group. Discrimination ratio = (time spent investigating changed object − time spent investigating unchanged object)/(time spent investigating changed object + time spent investigating unchanged object). Spontaneous Alternation percentage (%) = number of spontaneous alternations/(total number of arm entries − 2) × 100. One asterisk (*) representing *p*-value < 0.05 and two asterisks (**) representing *p*-value < 0.01.

Neurocognitive deficits are the hallmark of cancer-related cognitive impairment in cancer patients after therapy (Feiock et al., 2016; Santos and Pyter, 2018). To investigate neurocognitive impairment secondary to independent anti-cancer therapies such as doxorubicin and extracranial radiation therapy, a series of behavioral tests were performed. There was no significant difference in distance traveled between the control group and treatment groups (Figure 2A, Table 1). The treatment groups did not spend significantly more time in the middle of the open field maze compared to control mice (Figures 2B,D, Table 1). Additionally, the treatment groups did not spend significantly more time in the periphery of the open field maze compared to control mice (Figure 2C, Table 1).

**Table 1:** Behavioral test results.

	**Groups**	**Control**	**DOX**	**RT**	**DOX-RT**
**Open field test distance traveled**	**Mean (meters)**	26.26	26.64	21.61	30.98
	***P*-value**		0.9997	0.6648	0.6559
**Open field test time in the center of maze**	**Mean (seconds)**	67.24	66.81	45.43	61.34
	***P*-value**		>0.9999	0.5087	0.9814
**Open field test time in the periphery of maze**	**Mean (seconds)**	532.8	533.2	554.6	538.7
	***P*-value**		>0.9999	0.5087	0.9814
**Open field test percent time in center of maze**	**Mean** **(%)**	11.21	11.13	7.571	10.22
	***P*-value**		>0.9999	0.5087	0.9814
**Novel location recognition test discrimination ratio**	**Mean**	0.4441	0.09124	−0.05357	0.008871
	**Tukey *p*-value compared to control**		0.1042	0.0100**	0.0295*
	***P*-value compared to chance**	0.0018**	0.3269	0.6153	0.9318
**Novel object recognition test discrimination ratio**	**Mean**	0.4980	0.1764	0.2913	0.3388
	**Tukey *p*-value compared to control**		0.1460	0.5072	0.7050
	***P*-value compared to chance**	<0.0001**	0.2422	0.0167*	<0.0001**
**Y-maze spontaneous alternation**	**Mean (%)**	74.72	66.56	68.08	70.10
	***P*-value**		0.2343	0.4094	0.6986

*P*-values are compared to the Control Group in a one-way ANOVA with a post-hoc Tukey test. *P*-values for the novel location recognition test and novel object recognition test compared to chance (discrimination ratio of 0) in an unpaired *t*-test. Extended behavioral test results in Supplementary Table 1. One asterisk (*) representing *p*-value < 0.05 and two asterisks (**) representing *p*-value < 0.01.

The NLR test showed comparable hippocampal-dependent memory deficits in all treatment groups. Mice treated with RT or DOX-RT had a significantly lower discrimination ratio in the NLR test compared to control mice (Figure 2E). None of the treatment groups showed a preference for the novel location object (characterized by no significant difference compared to chance discrimination ratio of 0), demonstrating a hippocampal-dependent spatial learning and memory deficit (Table 1).

There were no significant differences in the discrimination ratio between treatment groups and control mice in the NOR test (Figure 2F). However, mice treated with DOX, contrary to the mice treated with DOX-RT or RT, failed to show a preference for the novel object (characterized by no significant difference compared to chance discrimination ratio of 0), demonstrating a decrease in hippocampal and cortical dependent recognition memory in this group (Table 1; Antunes and Biala, 2012; Cinalli et al., 2020). None of the treated mice had a demonstrable detrimental effect on working memory in the spontaneous alternation y-maze (Figure 2G, Table 1).

### Microgliosis and astrocytosis occur in all treatment groups but with anatomic variability

**Figure 3:**
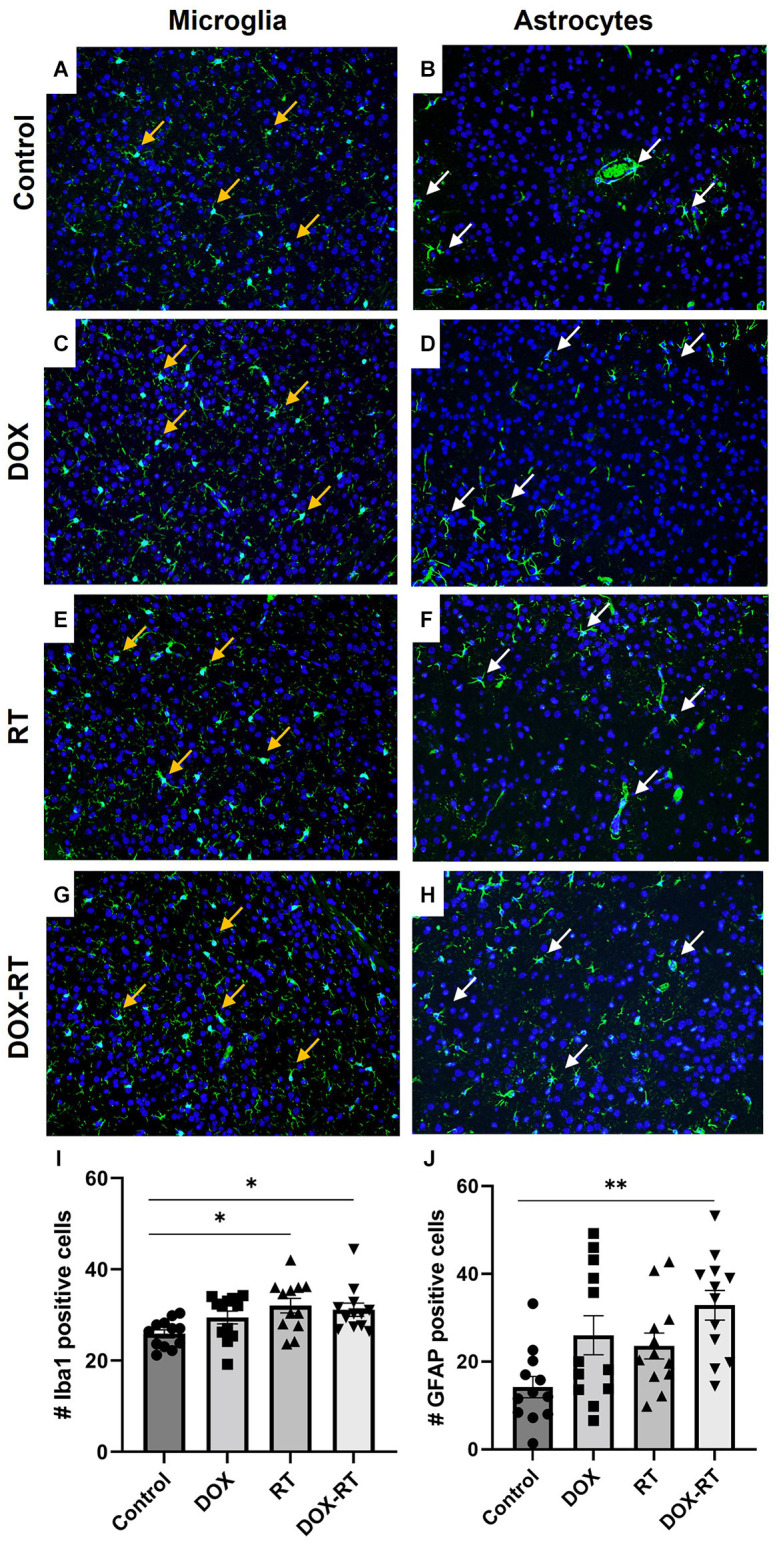
Cancer therapy causes microgliosis and astrocytosis in the caudal cortex. **(A–H)** Iba1+ microglia **(A,C,E,G)** are identified with orange arrows and GFAP+ astrocytes **(B,D,F,H)** are highlighted with white arrows. Representative images depict microglia and astrocytes from the caudal cortex in control mice **(A,B)** and mice treated with DOX **(C,D)**, RT **(E,F)** or DOX-RT **(G,H)**. Images shown are at 200× magnification. **(I)** Bar graph of Iba1+ microglia across treatment groups. Data represent the mean and SEM evaluated from four to five consecutive images per mouse (*n* = 12 per group). Mice treated with RT or DOX-RT had significantly more Iba1+ microglia compared to control mice. **(J)** Bar graph of GFAP+ astrocytes across treatment groups. Data represent the mean and SEM evaluated from four to five consecutive images per mouse (*n* = 12 per group). Mice treated with DOX-RT had significantly more GFAP+ astrocytes compared to control mice. DOX, doxorubicin group; RT, hindlimb radiation group; DOX-RT, doxorubicin and hindlimb radiation group. One asterisk (*) representing *p*-value < 0.05 and two asterisks (**) representing *p*-value < 0.01.

**Figure 4:**
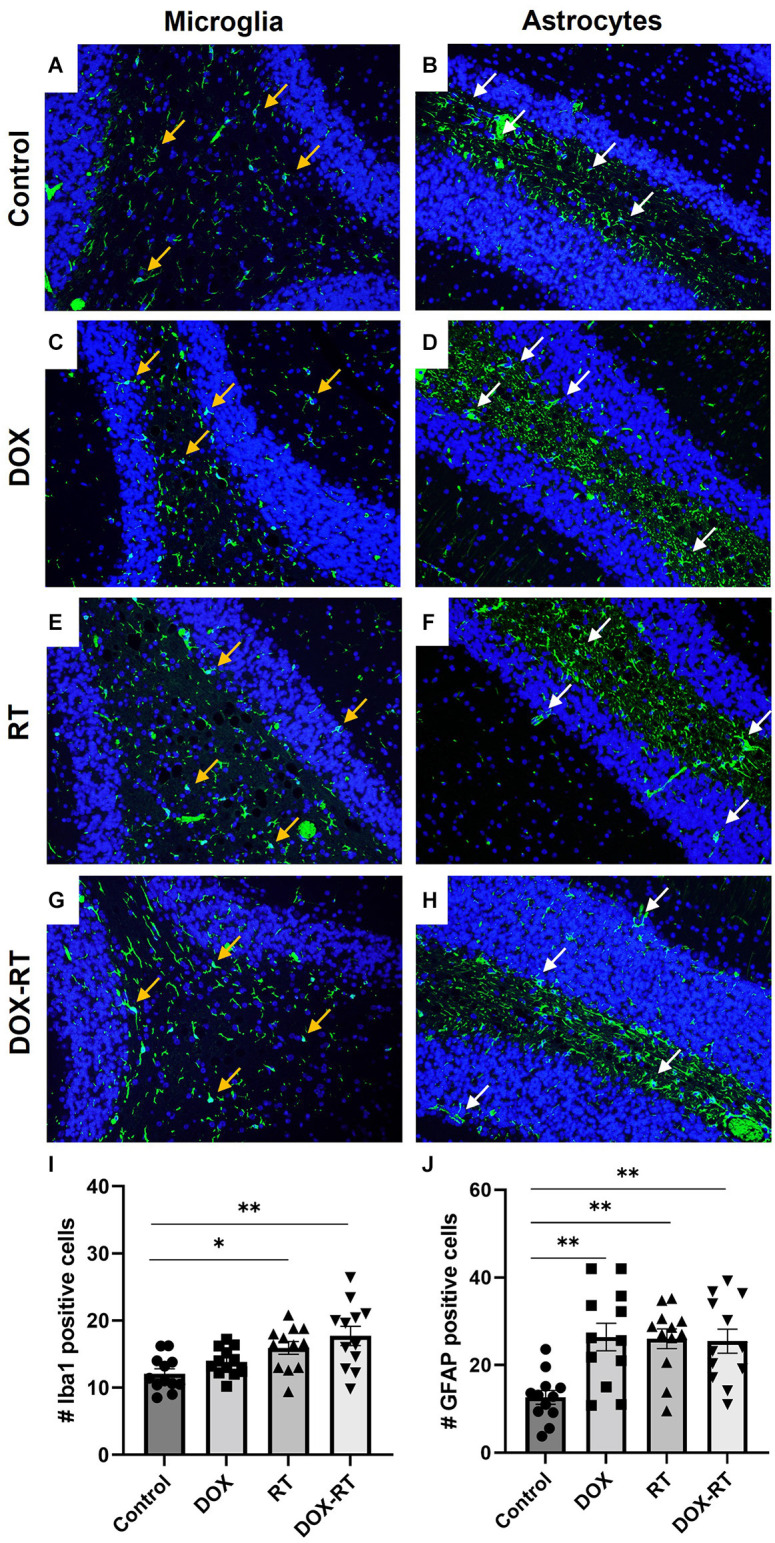
Cancer therapy causes microgliosis and astrocytosis in the cerebellum. **(A–H)** Iba1+ microglia **(A,C,E,G)** are identified with orange arrows and GFAP+ astrocytes **(B,D,F,H)** are shown with white arrows. Representative images depict microglia and astrocytes from the cerebellum in control mice **(A,B)** and mice treated with DOX **(C,D)**, RT **(E,F)** or DOX-RT **(G,H)**. Images shown are at 200× magnification. **(I)** Bar graph of Iba1+ microglia across treatment groups. Data represent the mean and SEM evaluated from four to five consecutive images per mouse (*n* = 12 per group). Mice treated with RT or DOX-RT had significantly more Iba1+ microglia compared to control mice. **(J)** Bar graph of GFAP+ astrocytes across treatment groups. Data represent the mean and SEM evaluated from four to five consecutive images per mouse (*n* = 12 per group). All treated mice had significantly more GFAP+ astrocytes compared to control mice. DOX, doxorubicin group; RT, hindlimb radiation group; DOX-RT, doxorubicin and hindlimb radiation group. One asterisk (*) representing *p*-value < 0.05 and two asterisks (**) representing *p*-value < 0.01.

**Figure 5:**
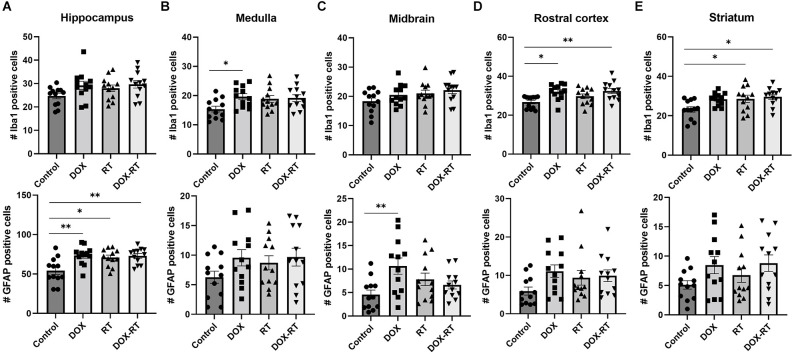
Cancer therapy causes multifocal microgliosis and astrocytosis. **(A–E)** Bar graphs depicting Iba1+ microglia (top row) and GFAP+ astrocytes (bottom row) are shown from the hippocampus **(A)**, medulla **(B)**, midbrain **(C)**, rostral cortex **(D)**, and striatum **(E)**. Control mice were compared to mice treated with DOX, RT, or DOX-RT. All three treatment groups had significant increases in GFAP+ astrocytes compared to control mice in the hippocampus **(A)**. Mice treated with DOX had more Iba1+ microglia compared to control mice in the medulla **(B)** and more GFAP+ astrocytes in the midbrain **(C)**. Mice treated with DOX or DOX-RT mice had significantly more Iba1+ microglia compared to control mice in the rostral cortex **(D)**. Mice treated with RT or DOX-RT mice had significantly more Iba1+ microglia compared to control mice in the striatum **(E)**. Data represent the mean and SEM evaluated from four to five consecutive images per mouse (*n* = 12 per group). DOX, doxorubicin group; RT, hindlimb radiation group; DOX-RT, doxorubicin and hindlimb radiation group. One asterisk (*) representing *p*-value < 0.05 and two asterisks (**) representing *p*-value < 0.01.

Significant changes in the number of activated microglia and/or astrocytes occurred in specific anatomic locations unique to each treatment group. In the caudal cortex, RT and DOX-RT mice had significantly more Iba1+ microglia compared to control mice (Figures 3A,C,E,G,I, Supplementary Table 2) and DOX-RT mice had significantly more GFAP+ astrocytes compared to control mice (Figures 3B,D,F,H,J, Supplementary Table 2). In the cerebellum, RT and DOX-RT mice had significantly more Iba1+ microglia compared to control mice (Figures 4A,C,E,G,I, Supplementary Table 2). All three treatment groups had significantly more GFAP+ astrocytes compared to control mice in the cerebellum (Figures 4B,D,F,H,J, Supplementary Table 2) and hippocampus (Figure 5A, Supplementary Table 2). There was no significant difference in hippocampal microglia number between groups (Figure 5A, Supplementary Table 2). In the medulla, DOX mice had significantly more Iba1+ microglia compared to control mice, but this was not seen in the DOX-RT mice (Figure 5B, Supplementary Table 2). In the midbrain, there was a significant increase in GFAP+ astrocytes in DOX mice compared to the control group (Figure 5C, Supplementary Table 2). The DOX and DOX-RT mice had significantly more Iba1+ microglia in the rostral cortex compared to controls (Figure 5D, Supplementary Table 2) and the RT and DOX-RT mice had more Iba1+ microglia in the striatum compared to controls (Figure 5E, Supplementary Table 2). No other significant differences were detected (Figure 5).

### Doxorubicin, but not extracranial irradiation, increases IL-6 cytokine concentrations in the peripheral blood 16 days following treatment

At the time of euthanasia, mice treated with DOX had significantly greater plasma concentration of IL-6 compared to mice treated with RT or saline (Supplementary Figure 2). This increased IL-6 was not present in mice treated with DOX-RT. There was no significant difference in plasma levels of IL-10, IL-12p70, IFN-γ, MCP-1, and TNF between groups.

## Discussion

Cognitive impairment resulting from chemotherapy and/or radiation therapy reduces the quality of life in cancer survivors (Christie et al., 2012; Mantos and Johnston, 2019). To date, mouse models that investigate the mechanisms by which cancer treatment negatively affects cognition have focused on chemotherapy (Santos and Pyter, 2018; Mantos and Johnston, 2019). Despite its well-documented bystander effects (both local and distant) and the frequency with which its prescribed (approximately 50% of patients with solid tumors), the contribution of radiation treatment (alone or concurrently with chemotherapy) to CRCI is poorly understood (Feiock et al., 2016). This is particularly notable, given that an estimated 4.2 million radiation-treated cancer survivors will be under care in the US by 2030 (Bryant et al., 2017). Our laboratory was the first to demonstrate that hindlimb irradiation causes widespread distant gliosis. However, the impact of this inflammation on cognition was not evaluated (Feiock et al., 2016). The results of this study, affirm and expand upon these previous observations by demonstrating both neurocognitive impairment and an increased number of activated microglia and astrocytes (components of neuroinflammation) following treatment with DOX, RT, or DOX-RT. Notably, these findings were generated in a strain of mice distinct from those in our previous work, which supports the generalizability of these findings (Feiock et al., 2016).

Using the NLR test, we demonstrated hippocampal-dependent deficits in spatial memory in all treatment groups. Although such deficits have been reported in previous rodent studies following treatment with numerous chemotherapeutics including doxorubicin, methotrexate, 5-fluorouracil, docetaxel, topotecan, oxaliplatin, cyclophosphamide or cisplatin, this study is the first to document such deficits as a distant bystander effect of RT (Christie et al., 2012; Kitamura et al., 2015; Seigers et al., 2015; Mantos and Johnston, 2019). Coincident with the neurocognitive deficits, we also observed a significant hippocampal astrocytosis across all three treatment groups. There was a trend in all treatment groups of more hippocampal-activated microglia. Based upon the neurocognitive and neuroinflammatory pathology observed in our DOX-treated mice and the results of previous studies, which have demonstrated hippocampal microgliosis and astrocytosis and neurocognitive deficits in rats, mice, and humans treated with systemic chemotherapy, we propose that the distant brain effects of doxorubicin treatment are a consequence of systemic inflammation (El-Agamy et al., 2018; El-Derany and Noureldein, 2021; Gibson and Monje, 2021; Ongnok et al., 2021; Schroyen et al., 2021; Wang et al., 2021).

Microglia and astrocytes promote neuronal survival but when activated these glial cells can cause neuronal damage subsequently leading to neurocognitive deficits (Liddelow et al., 2017; Gibson and Monje, 2021). The activation of microglia induced by cancer therapy has been implicated in causing the dysregulation of neuronal signaling by blocking neurogenesis, aberrant pruning of dendrites and axonal terminals, and impeding myelin plasticity (Gibson and Monje, 2021). Microglia activation can also lead to the stimulation of neurotoxic astrocyte reactivity, which has been seen in multiple human neurodegenerative diseases (Liddelow et al., 2017; Gibson and Monje, 2021). The activation of neurotoxic astrocytes can induce neuron and oligodendrocytes death through the secretion of neurotoxins and blocking oligodendrocytes precursor cells proliferation and differentiation which can lead to neurocognitive deficits (Liddelow et al., 2017).

The DOX mice demonstrated a deficit in recognition memory, which involves the hippocampus and perirhinal cortex which was expected based on prior studies in mice and rats (Seigers et al., 2015; Ramalingayya et al., 2017; Barry et al., 2018; Keeney et al., 2018; Cinalli et al., 2020). In our study, this deficit was associated with significant rostral cortical microgliosis, hippocampal astrocytosis, and increased plasma IL-6 concentration. Surprisingly, we did not observe recognition memory deficits in the DOX-RT mice despite similarly significant rostral cortical and hippocampal microgliosis and astrocytosis. Based on our analysis, we speculate that the increased plasma IL-6 concentration in the DOX mice underlies this difference. This speculation aligns with one report of human cancer patients, in which increased circulating IL-6 was associated with cognitive impairment (Lange et al., 2019). While radiation typically induces an early increase in systemic IL-6, it is possible that radiation orchestrated a different temporal cytokine profile, even when combined with doxorubicin (Chen et al., 2001; Ao et al., 2009; Kiprian et al., 2018). Our results are supported by other work demonstrating that doxorubicin, but not radiation, leads to detectable recognition memory impairment (Seigers et al., 2015; McGinnis et al., 2017; Ramalingayya et al., 2017; Barry et al., 2018; Keeney et al., 2018; Cinalli et al., 2020).

None of our treatment groups demonstrated locomotor function deficits as assessed by the open field test. This was surprising when considering the significant microgliosis in the rostral cortex, a brain region which includes the motor cortex region in both the DOX and DOX-RT mice. Our results are consistent with previous rodent doxorubicin studies and likely reflect the numerous non-neurocognitive factors, including spinal cord status, motor output, and circadian rhythm that contribute to effective locomotion (Aziriova et al., 2014; Tatem et al., 2014). Although anxiety is reported to influence locomotion, none of the mice in our study demonstrated anxiety-like behavior, which is similar to prior doxorubicin and extracranial radiation treatment work (Seigers et al., 2015; McGinnis et al., 2017).

Despite identifying significant hippocampal astrocytosis in all treatment groups and significant prefrontal cortical microgliosis in the DOX mice, a working memory deficit was not observed. The spontaneous alternation y-maze tests multiple regions of the brain that contribute to working memory, including the prefrontal cortex, hippocampus, basal forebrain, dorsomedial thalamus, basal ganglia, and vestibular circuitry (Bizon et al., 2012; Kraeuter et al., 2019). Our results differ from prior studies demonstrating that doxorubicin-treated rats had a significantly lower percent of spontaneous alternations than control rats at 7, 9 or 10 days post-treatment (El-Derany and Noureldein, 2021; Ibrahim et al., 2021; Shaker et al., 2021). This difference may be a reflection of the later timing of neurocognitive testing in our study.

In a previous study, BALB/c mice treated with a single hindlimb dose of 16 Gy radiation given with 320 kV demonstrated significant brain gliosis characterized by widespread microgliosis and astrocytosis that persisted at least 30 days post-irradiation (Feiock et al., 2016). In this current study, irradiated SKH1 mice treated with a single hindlimb dose of 20 Gy given with 6 MeV were also found to have significant brain gliosis. Both groups of irradiated mice had significant microgliosis in the striatum and significant microgliosis and astrocytosis in the cerebellum, while neither strain developed midbrain inflammation (Feiock et al., 2016). However, microgliosis was observed in the caudal cortex of the irradiated SKH1 mice, but this was not observed in the BALB/c mice (Feiock et al., 2016). Additionally, BALB/c mice had significant microgliosis in the medulla at 3 and 30 days post-treatment, but this was not observed in SKH1 mice (Feiock et al., 2016). Finally, SKH1 mice, but not BALB/c mice, demonstrated hippocampal astrocytosis post-irradiation (Feiock et al., 2016). These differences could reflect strain-dependent differences in radiosensitivity, differences related to RT delivery (i.e., different energies and dose rates between radiation delivery units), and/or differences in timing of assessment. Importantly, although the demonstration of RT-induced gliosis across two strains of mice reveals the potential generalizability of this phenomenon, these distinct, brain region-specific results affirm the importance of systematic model characterization.

One potential mechanism leading to the bystander microgliosis and astrocytosis following hindlimb radiation is through the in-field production, and systemic trafficking, of pro-inflammatory mediators (TNF-α, IL-1, IL-6, and reactive oxygen species), particularly as these molecules are known to be produced by irradiated cells and can cross the blood-brain barrier (Schaue et al., 2012; Morgan and Sowa, 2015; Feiock et al., 2016; Santos and Pyter, 2018; Wang et al., 2018). Reactive oxygen species, TNF-α, and IL-1 have been shown to cause pathogenesis including neuroinflammation that can lead to neurodegeneration in the brain after radiation (Schaue et al., 2012; Shabab et al., 2017). Although, there were no significant increases in circulating pro-inflammatory cytokines 16 days post-radiation treatment, it is possible that the peak in cytokine levels occurred prior to histologic injury and earlier assessments in the future may reveal additional changes.

Significant microgliosis and/or astrocytosis was detected in the cerebellum, hippocampus, medulla, midbrain, and rostral cortex, of the DOX mice. These findings align with previous work demonstrating doxorubicin-induced microgliosis and astrocytosis (Santos and Pyter, 2018; Ali et al., 2020; Ongnok et al., 2021; Wang et al., 2021). Similar to the RT-treated mice, one potential mechanism for microgliosis and astrocytosis is the systemic trafficking of pro-inflammatory cytokines including TNF-α (Tangpong et al., 2006; Ongnok et al., 2020). The impact of circulating TNF-α on the brain is well-characterized, including its ability to directly activate microglia and astrocytes; increase local production of additional TNF-α; induce glutamate-mediated neurotoxicity, and directly damage neurons through mitochondrial dysfunction (Tangpong et al., 2006; Ongnok et al., 2020). Similar to the RT mice, it is possible that increases in circulating TNF-α were not detected in the DOX mice due to the sampling strategy.

In our study, DOX, and DOX-RT-treated mice had significantly decreased body weight change compared to RT or control mice. This is not unexpected and likely reflects known doxorubicin-related gastrointestinal toxicity (Seigers et al., 2015; Lyu et al., 2021). The results of the open field test do not support that these mice are eating less due to mobility impairment. On necropsy, no significant peritoneal or omental toxicity was noted to suggest IP administration directly contributed to weight loss, but it is possible that subacute peritoneal irritation contributed to weight loss. Further investigation will be needed to determine the cause of the lack of weight gain in these mice. No other toxicities were noted during the duration of the study.

Limitations of this study will be addressed in future work. First, only a single treatment of doxorubicin and/or radiation was used. This abbreviated treatment plan allowed for the detection of measurable cognitive impairment and gliosis and further studies can evaluate the intensity of changes following cumulative dosing schemes. Circulating cytokines, and the number of activated microglia and astrocytes were only evaluated at a single time point in this mouse study, and earlier changes may better reflect impending cognitive deficits. Serial blood and tissue sampling in rodent models is challenging and often requires the use of more animals. Additionally, it will be important to contrast our findings in female SKH1 mice to male SKH1 mice, as well as in young mice compared to old mice. Men are also at risk for CRCI, although they are vastly under-represented in the existing literature given the prevalence of CRCI in breast cancer patients. Future similar or different findings between sexes of mice may highlight unique pathways that contribute to the pathogenesis of CRCI. Our data establish a framework for studying the neurocognitive effects of anticancer treatments in a unique SKH1 mouse model. Results highlight that significant and unintended changes occur in the brain following a single treatment of doxorubicin and/or irradiation.

## Conclusion

Doxorubicin and radiation therapy were associated with comparable hippocampal memory deficits yet distinctive anatomic patterns of astrocytosis and microgliosis in SKH1 mice. This study sheds light on the similarities in cognitive impairment following local radiation therapy and/or systemic chemotherapy. Further work is needed to identify potential strategies to minimize cognitive deficits in cancer survivors.

## Data Availability Statement

The raw data supporting the conclusions of this article will be made available by the authors, without undue reservation.

## Ethics Statement

The animal study was reviewed and approved by University of Minnesota Institutional Animal Care and Use Committee (IACUC).

## Author Contributions

KD-D, JL, EL, and DS designed, performed experiments, and analyzed data. AR analyzed data. KD-D, JL, and DS secured funding. All authors contributed to the article and approved the submitted version.
