# Supplementary material for: Cancer treatment induces neuroinflammation and behavioral deficits in mice
Source: Front Behav Neurosci. 2023 Jan 9;16:1067298. doi: 10.3389/fnbeh.2022.1067298 (PMC9868853; doi:10.3389/fnbeh.2022.1067298)
Supplement: Supplementary file 1 [file Table_1.DOCX]

**Supplement Table 1: Extended behavioral test results.** P-values are compared to the Control Group in a one-way ANOVA with a post-hoc Tukey test. P-values for the novel location recognition test and novel object recognition test compared to chance (discrimination ratio of 0) in an unpaired t-test. SEM=standard error of mean, DF= degrees of freedom. One asterisk (*) representing p value < 0.05 and two asterisks (**) representing p-value < 0.01.

|  | **Groups** | **Control** | **DOX** | **RT** | **DOX-RT** |
| --- | --- | --- | --- | --- | --- |
| **Open field test distance traveled** | **Mean (meters)** | 26.26 | 26.64 | 21.61 | 30.98 |
|  | **SEM** | 3.761 | 2.025 | 2.207 | 3.157 |
|  | **Statistical test** | one-way ANOVA with a post-hoc Tukey test | | | |
|  | **Tukey p-value** |  | 0.9997 | 0.6648 | 0.6559 |
|  | **F-value** | 1.770 | | | |
|  | **P-value** | 0.1668 | | | |
|  | **DF** | 47 | | | |
|  | **Number of samples** | 12 | 12 | 12 | 12 |
| **Open field test time in the center of maze** | **Mean (seconds)** | 67.24 | 66.81 | 45.43 | 61.34 |
|  | **SEM** | 12.15 | 8.483 | 11.90 | 11.29 |
|  | **Statistical test** | one-way ANOVA with a post-hoc Tukey test | | | |
|  | **Tukey p-value** |  | >0.9999 | 0.5087 | 0.9814 |
|  | **F-value** | 0.8536 | | | |
|  | **P-value** | 0.4723 | | | |
|  | **DF** | 47 | | | |
|  | **Number of samples** | 12 | 12 | 12 | 12 |
| **Open field test time in the periphery of maze** | **Mean (seconds)** | 532.8 | 533.2 | 554.6 | 538.7 |
|  | **SEM** | 12.15 | 8.483 | 11.90 | 11.29 |
|  | **Statistical test** | one-way ANOVA with a post-hoc Tukey test | | | |
|  | **Tuckey p-value** |  | >0.9999 | 0.5087 | 0.9814 |
|  | **F-value** | 0.8536 | | | |
|  | **P-value** | 0.4723 | | | |
|  | **DF** | 47 | | | |
|  | **Number of samples** | 12 | 12 | 12 | 12 |
| **Open field test percent time in center of maze** | **Mean (%)** | 11.21 | 11.13 | 7.571 | 10.22 |
|  | **SEM** | 2.025 | 1.414 | 1.983 | 1.882 |
|  | **Statistical test** | one-way ANOVA with a post-hoc Tukey test | | | |
|  | **Tukey p-value** |  | >0.9999 | 0.5087 | 0.9814 |
|  | **F-value** | 0.8536 | | | |
|  | **P-value** | 0.4723 | | | |
|  | **DF** | 47 | | | |
|  | **Number of samples** | 12 | 12 | 12 | 12 |
| **Novel location recognition test discrimination ratio** | **Mean** | 0.4441 | 0.09124 | -0.05357 | 0.008871 |
|  | **SEM** | 0.1250 | 0.09100 | 0.1051 | 0.1024 |
|  | **Statistical Test** | one-way ANOVA with a post-hoc Tukey test | | | |
|  | **Tukey p-value compared to control** |  | 0.1042 | 0.0100** | 0.0295* |
|  | **F-value** | 4.353 | | | |
|  | **P-value** | 0.0090** | | | |
|  | **DF** | 47 | | | |
|  | **Statistical test** | Unpaired t test | | | |
|  | **P-value compared to chance** | 0.0018** | 0.3269 | 0.6153 | 0.9318 |
|  | **t-value** | 3.554 | 1.003 | 0.5097 | 0.08661 |
|  | **DF** | 22 | 22 | 22 | 22 |
|  | **Number of samples** | 12 | 12 | 12 | 12 |
| **Novel object recognition test discrimination ratio** | **Mean** | 0.4980 | 0.1764 | 0.2913 | 0.3388 |
|  | **SEM** | 0.06671 | 0.1468 | 0.1124 | 0.07123 |
|  | **Statistical test** | one-way ANOVA with a post-hoc Tukey test | | | |
|  | **Tukey p-value compared to control** |  | 0.1460 | 0.5072 | 0.7050 |
|  | **F-value** | 1.627 | | | |
|  | **P-value** | 0.1967 | | | |
|  | **DF** | 47 | | | |
|  | **Statistical test** | Unpaired t test | | | |
|  | **P-value compared to chance** | <0.0001** | 0.2422 | 0.0167* | <0.0001** |
|  | **t-value** | 7.466 | 1.202 | 2.591 | 4.756 |
|  | **DF** | 22 | 22 | 22 | 22 |
|  | **Number of samples** | 12 | 12 | 12 | 12 |
| **Y-maze spontaneous alternation** | **Mean (%)** | 74.72 | 66.56 | 68.08 | 70.10 |
|  | **SEM** | 2.961 | 3.201 | 2.740 | 3.086 |
|  | **Statistical test** | one-way ANOVA with a post-hoc Tukey test | | | |
|  | **Tukey p-value** |  | 0.2343 | 0.4094 | 0.6986 |
|  | **F-value** | 1.394 | | | |
|  | **P-value** | 0.2571 | | | |
|  | **DF** | 47 | | | |
|  | **Number of samples** | 12 | 12 | 12 | 12 |

**Supplement Table 2: Histology results** P-values are compared to the Control Group in a one-way ANOVA with a post-hoc Tukey test. SEM=standard error of mean, DF- degrees of freedom. One asterisk (*) representing p value < 0.05 and two asterisks (**) representing p-value < 0.01.

|  |  | **Groups** | **Control** | **DOX** | | **RT** | | **DOX-RT** | |
| --- | --- | --- | --- | --- | --- | --- | --- | --- | --- |
| **Caudal cortex** | **Iba1** | **Mean (# of positive cells)** | 25.87 | 29.43 | | 32.03 | | 31.12 | |
|  |  | **SEM** | 0.8743 | 1.405 | | 1.605 | | 1.434 | |
|  |  | **Statistical test** | one-way ANOVA with a post-hoc Tukey test | | | | | | |
|  |  | **Tukey p-value** |  | 0.2607 | | 0.0127* | | 0.0428* | |
|  |  | **F-value** | 4.015 | | | | | | |
|  |  | **P-value** | 0.0131* | | | | | | |
|  |  | **DF** | 47 | | | | | | |
|  | **GFAP** | **Mean (# of positive cells)** | 14.20 | | 26.03 | | 23.58 | | 32.85 |
|  |  | **SEM** | 2.424 | | 4.451 | | 2.959 | | 3.382 |
|  |  | **Statistical test** | one-way ANOVA with a post-hoc Tukey test | | | | | | |
|  |  | **Tukey p-value** |  | | 0.0788 | | 0.2194 | | 0.0018** |
|  |  | **F-value** | 5.189 | | | | | | |
|  |  | **P-value** | 0.0037** | | | | | | |
|  |  | **DF** | 47 | | | | | | |
|  |  | **Number of samples** | 12 | 12 | | 12 | | 12 | |
| **Cerebellum** | **Iba1** | **Mean (# of positive cells)** | 12.07 | 13.85 | | 15.93 | | 17.72 | |
|  |  | **SEM** | 0.7472 | 0.6026 | | 0.9472 | | 1.420 | |
|  |  | **Statistical test** | one-way ANOVA with a post-hoc Tukey test | | | | | | |
|  |  | **Tukey p-value** |  | 0.5772 | | 0.0376* | | 0.0010** | |
|  |  | **F-value** | 6.296 | | | | | | |
|  |  | **P-value** | 0.0012** | | | | | | |
|  |  | **DF** | 47 | | | | | | |
|  | **GFAP** | **Mean (# of positive cells)** | 12.65 | | 26.42 | | 26.02 | | 25.48 |
|  |  | **SEM** | 1.590 | | 3.165 | | 2.241 | | 2.764 |
|  |  | **Statistical test** | one-way ANOVA with a post-hoc Tukey test | | | | | | |
|  |  | **Tukey p-value** |  | | 0.0019** | | 0.0027** | | 0.0041** |
|  |  | **F-value** | 7.064 | | | | | | |
|  |  | **P-value** | 0.0006** | | | | | | |
|  |  | **DF** | 47 | | | | | | |
|  |  | **Number of samples** | 12 | 12 | | 12 | | 12 | |
| **Hippocampus** | **Iba1** | **Mean (# of positive cells)** | 24.68 | 29.22 | | 28.00 | | 29.70 | |
|  |  | **SEM** | 1.165 | 1.773 | | 1.411 | | 1.602 | |
|  |  | **Statistical test** | one-way ANOVA with a post-hoc Tukey test | | | | | | |
|  |  | **Tukey p-value** |  | 0.1589 | | 0.4113 | | 0.1003 | |
|  |  | **F-value** | 2.261 | | | | | | |
|  |  | **P-value** | 0.0946 | | | | | | |
|  |  | **DF** | 47 | | | | | | |
|  | **GFAP** | **Mean (# of positive cells)** | 54.17 | | 73.43 | | 70.75 | | 72.90 |
|  |  | **SEM** | 4.681 | | 3.378 | | 3.155 | | 2.880 |
|  |  | **Statistical test** | one-way ANOVA with a post-hoc Tukey test | | | | | | |
|  |  | **Tukey p-value** |  | | 0.0024** | | 0.0110* | | 0.0033** |
|  |  | **F-value** | 6.523 | | | | | | |
|  |  | **P-value** | 0.0010** | | | | | | |
|  |  | **DF** | 47 | | | | | | |
|  |  | **Number of samples** | 12 | 12 | | 12 | | 12 | |
| **Medulla** | **Iba1** | **Mean (# of positive cells)** | 15.39 | 19.63 | | 18.87 | | 19.17 | |
|  |  | **SEM** | 0.9852 | 1.029 | | 1.137 | | 1.135 | |
|  |  | **Statistical test** | one-way ANOVA with a post-hoc Tukey test | | | | | | |
|  |  | **Tukey p-value** |  | 0.0377* | | 0.1163 | | 0.0762 | |
|  |  | **F-value** | 3.262 | | | | | | |
|  |  | **P-value** | 0.0302* | | | | | | |
|  |  | **DF** | 47 | | | | | | |
|  | **GFAP** | **Mean (# of positive cells)** | 6.267 | | 9.550 | | 8.700 | | 9.667 |
|  |  | **SEM** | 1.021 | | 1.379 | | 1.193 | | 1.502 |
|  |  | **Statistical test** | one-way ANOVA with a post-hoc Tukey test | | | | | | |
|  |  | **Tukey p-value** |  | | 0.2851 | | 0.5448 | | 0.2564 |
|  |  | **F-value** | 1.506 | | | | | | |
|  |  | **P-value** | 0.2261 | | | | | | |
|  |  | **DF** | 47 | | | | | | |
|  |  | **Number of samples** | 12 | 12 | | 12 | | 12 | |
| **Midbrain** | **Iba1** | **Mean (# of positive cells)** | 18.32 | 20.48 | | 21.00 | | 22.10 | |
|  |  | **SEM** | 1.110 | 1.034 | | 1.124 | | 1.172 | |
|  |  | **Statistical test** | one-way ANOVA with a post-hoc Tukey test | | | | | | |
|  |  | **Tukey p-value** |  | 0.5189 | | 0.3319 | | 0.0903 | |
|  |  | **F-value** | 2.046 | | | | | | |
|  |  | **P-value** | 0.1213 | | | | | | |
|  |  | **DF** | 47 | | | | | | |
|  | **GFAP** | **Mean (# of positive cells)** | 4.567 | | 10.63 | | 7.800 | | 6.617 |
|  |  | **SEM** | 0.9547 | | 1.734 | | 1.341 | | 0.8501 |
|  |  | **Statistical test** | one-way ANOVA with a post-hoc Tukey test | | | | | | |
|  |  | **Tukey p-value** |  | | 0.0080** | | 0.2862 | | 0.6657 |
|  |  | **F-value** | 3.986 | | | | | | |
|  |  | **P-value** | 0.0135* | | | | | | |
|  |  | **DF** | 47 | | | | | | |
|  |  | **Number of samples** | 12 | 12 | | 12 | | 12 | |
| **Rostral cortex** | **Iba1** | **Mean (# of positive cells)** | 26.76 | 32.03 | | 29.72 | | 32.43 | |
|  |  | **SEM** | 0.9055 | 1.159 | | 1.197 | | 1.362 | |
|  |  | **Statistical test** | one-way ANOVA with a post-hoc Tukey test | | | | | | |
|  |  | **Tukey p-value** |  | 0.0133* | | 0.2908 | | 0.0068** | |
|  |  | **F-value** | 4.996 | | | | | | |
|  |  | **P-value** | 0.0046** | | | | | | |
|  |  | **DF** | 47 | | | | | | |
|  | **GFAP** | **Mean (# of positive cells)** | 5.917 | | 11.10 | | 9.383 | | 9.917 |
|  |  | **SEM** | 1.049 | | 1.629 | | 1.910 | | 1.517 |
|  |  | **Statistical test** | one-way ANOVA with a post-hoc Tukey test | | | | | | |
|  |  | **Tukey p-value** |  | | 0.1016 | | 0.4038 | | 0.2797 |
|  |  | **F-value** | 2.044 | | | | | | |
|  |  | **P-value** | 0.1215 | | | | | | |
|  |  | **DF** | 47 | | | | | | |
|  |  | **Number of samples** | 12 | 12 | | 12 | | 12 | |
| **Striatum** | **Iba1** | **Mean (# of positive cells)** | 23.20 | 28.37 | | 28.60 | | 29.61 | |
|  |  | **SEM** | 1.386 | 0.9397 | | 1.808 | | 1.331 | |
|  |  | **Statistical test** | one-way ANOVA with a post-hoc Tukey test | | | | | | |
|  |  | **Tukey p-value** |  | 0.0574 | | 0.0440* | | 0.0119* | |
|  |  | **F-value** | 4.228 | | | | | | |
|  |  | **P-value** | 0.0104* | | | | | | |
|  |  | **DF** | 47 | | | | | | |
|  | **GFAP** | **Mean (# of positive cells)** | 5.100 | | 8.467 | | 6.767 | | 8.800 |
|  |  | **SEM** | 0.7196 | | 1.452 | | 1.229 | | 1.411 |
|  |  | **Statistical test** | one-way ANOVA with a post-hoc Tukey test | | | | | | |
|  |  | **Tukey p-value** |  | | 0.2332 | | 0.7769 | | 0.1646 |
|  |  | **F-value** | 1.901 | | | | | | |
|  |  | **P-value** | 0.1434 | | | | | | |
|  |  | **DF** | 47 | | | | | | |
|  |  | **Number of samples** | 12 | 12 | | 12 | | 12 | |
