# Supplementary material for: Cancer treatment induces neuroinflammation and behavioral deficits in mice
Source: Front Behav Neurosci. 2023 Jan 9;16:1067298. doi: 10.3389/fnbeh.2022.1067298 (PMC9868853; doi:10.3389/fnbeh.2022.1067298)
Supplement: Supplementary file 2 [file Presentation_1.PPTX]

## Slide 1
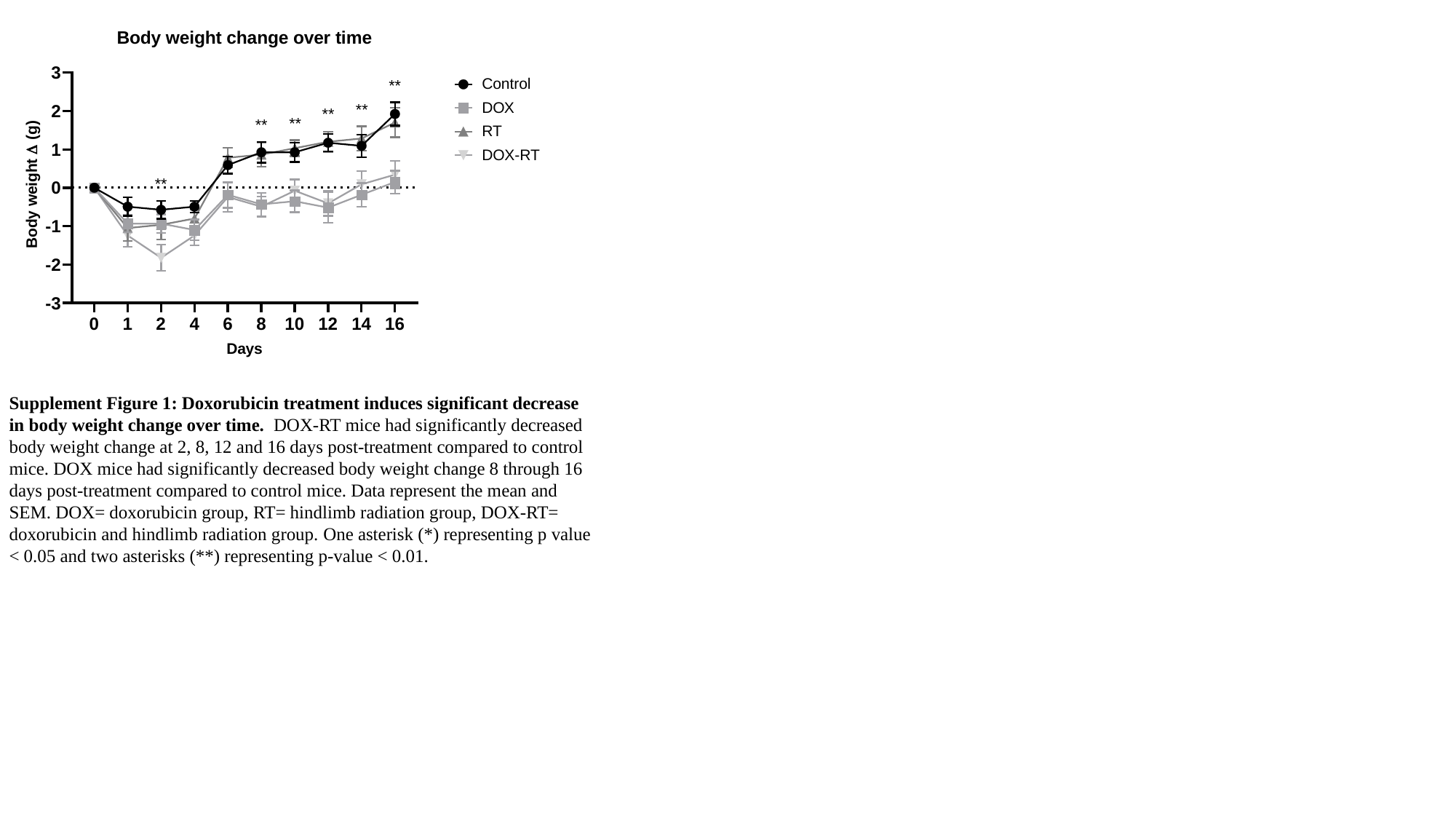

Supplement Figure 1: Doxorubicin treatment induces significant decrease in body weight change over time. DOX-RT mice had significantly decreased body weight change at 2, 8, 12 and 16 days post-treatment compared to control mice. DOX mice had significantly decreased body weight change 8 through 16 days post-treatment compared to control mice. Data represent the mean and SEM. DOX= doxorubicin group, RT= hindlimb radiation group, DOX-RT= doxorubicin and hindlimb radiation group. One asterisk (*) representing p value < 0.05 and two asterisks (**) representing p-value < 0.01.

## Slide 2
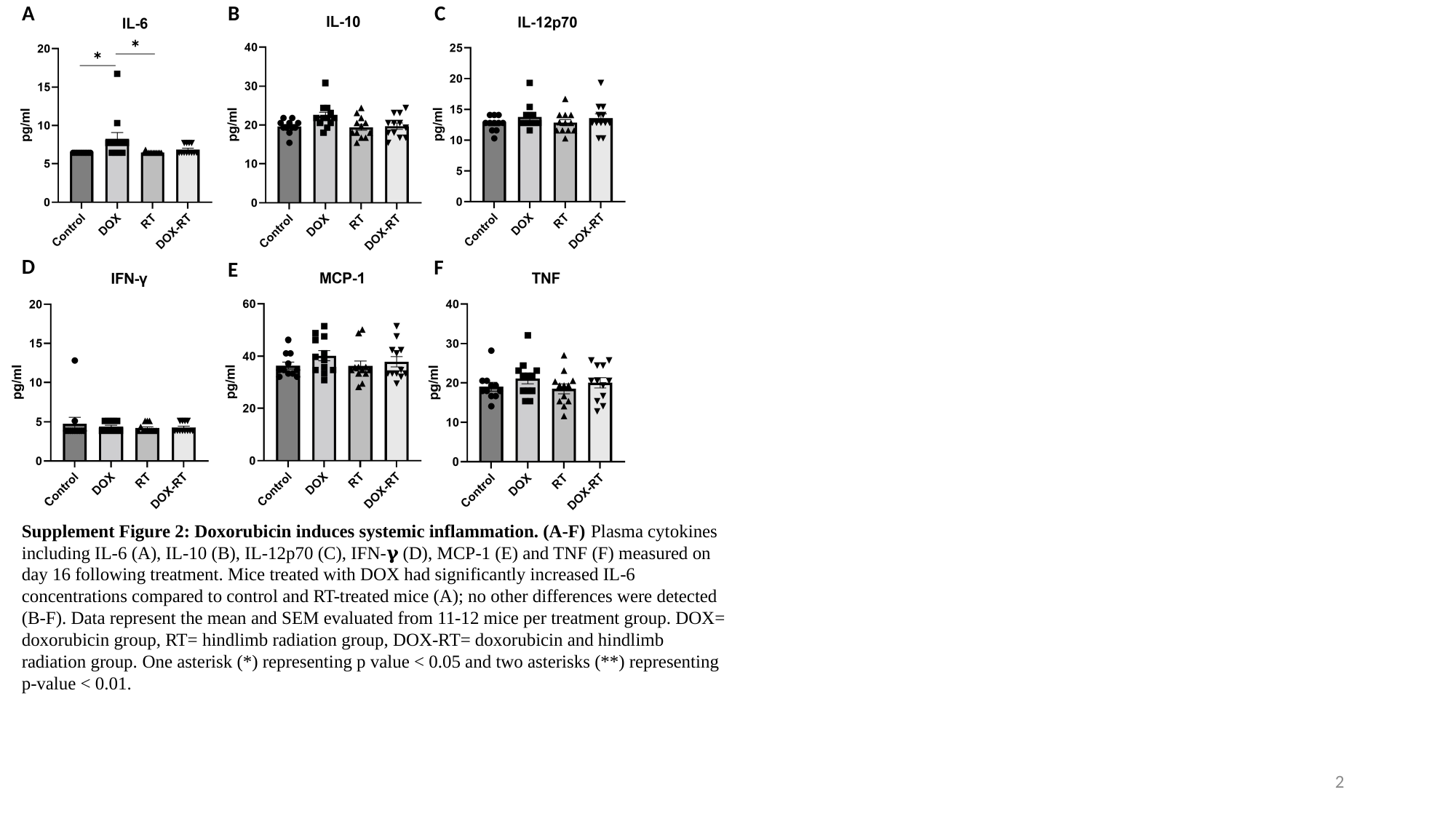

A
B
C
D
F
E
Supplement Figure 2: Doxorubicin induces systemic inflammation. (A-F) Plasma cytokines including IL-6 (A), IL-10 (B), IL-12p70 (C), IFN-𝛄 (D), MCP-1 (E) and TNF (F) measured on day 16 following treatment. Mice treated with DOX had significantly increased IL-6 concentrations compared to control and RT-treated mice (A); no other differences were detected (B-F). Data represent the mean and SEM evaluated from 11-12 mice per treatment group. DOX= doxorubicin group, RT= hindlimb radiation group, DOX-RT= doxorubicin and hindlimb radiation group. One asterisk (*) representing p value < 0.05 and two asterisks (**) representing p-value < 0.01.
2
